# The efficacy of recombinant human soluble thrombomodulin (rhsTM) treatment for acute exacerbation of idiopathic pulmonary fibrosis: a systematic review and meta-analysis

**DOI:** 10.1186/s12890-020-1092-3

**Published:** 2020-03-02

**Authors:** Hiroyuki Kamiya, Ogee Mer Panlaqui

**Affiliations:** 1 School of Population and Global Health, University of Western Australia, 35 Stirling Highway, Perth, Western Australia 6009 Australia; 2 Department of Intensive Care Medicine, Northern Hospital, 185 Cooper Street, Epping, Victoria 3076 Australia

**Keywords:** Recombinant human soluble thrombomodulin, Acute exacerbation, Idiopathic pulmonary fibrosis, Review, Meta-analysis

## Abstract

**Background:**

Acute exacerbation (AE) of idiopathic pulmonary fibrosis (IPF) is devastating with no established treatment. This phenomenon involves disordered coagulation and excessive inflammatory reactions. As recombinant human soluble thrombomodulin (rhsTM) possesses anti-coagulative and anti-inflammatory properties, the medicine is expected to improve the prognosis of the disease. The aim of this study was to summarize current evidence regarding benefits and harms of rhsTM treatment for AE of IPF.

**Method:**

Patients with AE of IPF were eligible for the review and all of the other types of interstitial pneumonias were excluded. The effect of rhsTM treatment on the outcomes such as all-cause mortality was estimated in comparison to conventional therapy. Primary studies of any design aside from a case report were reviewed. Electronic databases such as Medline and EMBASE were searched from 2002 through August 14, 2019. Two reviewers independently selected eligible reports and extracted relevant data. A risk of bias of individual studies was assessed similarly. Meta-analysis was conducted for univariate results if at least three studies were available for the same outcome.

**Result:**

Out of a total of 390 records identified, eight studies were first deemed eligible and four of them were finally focused for the review. Only one study was a prospective trial and a historical control was employed in all studies. An overall risk of bias was rated as serious in three out of four studies. A total of 169 subjects were included. Two out of three studies that reported 3-month all-cause mortality by univariate analysis demonstrated beneficial effects of rhsTM treatment and a pooled analysis demonstrated that rhsTM treatment improved 3-month all-cause mortality with a risk ratio of 0.50 (95% confidence interval (CI): 0.35–0.72). All two studies reporting multivariate results demonstrated that rhsTM treatment improved 3-month all-cause mortality with odds ratios of 0.21 (95% CI: 0.05–0.91) and 0.25 (95% CI: 0.09–0.68), respectively. There were no serious adverse events.

**Conclusion:**

The rhsTM treatment was demonstrated to improve 3-month all-cause mortality of AE of IPF with no serious adverse events. However, these findings should be interpreted with caution due to a small number of studies and serious risk of bias.

## Background

Idiopathic pulmonary fibrosis (IPF) is the most common type of interstitial pneumonia (IP) with unknown aetiology that is categorized as chronic fibrosing interstitial pneumonia (CFIP) alongside of idiopathic non-specific interstitial pneumonia (NSIP) [1]. IPF is a progressive disease and depicts the dismal clinical course with an estimated survival time of 2 to 3 years after its diagnosis [2]. The predominant cause of deaths of the disease is an acute exacerbation (AE), which is reported to be responsible for 40% of the cases [3]. AE of IPF was initially defined as acute respiratory worsening beyond its usually anticipated gradual progression triggered by no identifiable causes [4]. Subsequently, this overwhelming phenomenon was noted to be not unique to IPF but also complicate other types of IPs such as idiopathic NSIP, connective tissue disease-associated interstitial pneumonia (CTD-IP) and chronic hypersensitivity pneumonitis [5–7]. Although the frequency of AE and its prognosis varies depending on underlying IPs [5–7], IPF is the most prevalent among others [8], and the prognosis of AE of IPF is uniformly poor with 90-day mortality of 40 to 50% [9]. This devastating outcome is mostly because the pathogenesis of this phenomenon has yet to be fully elucidated and there is currently no established treatment that has been proved beneficial [10]. Therefore, it is urgent to develop a new therapeutic strategy to address substantial clinical burden caused by AE of IPF and improve the prognosis of the disease.

Recombinant human soluble thrombomodulin (rhsTM) is a relatively new medicine that uses the technology of cloning [11]. It consists of an extracellular domain of native thrombomodulin, a transmembrane glycoprotein expressed on the endothelial cell surface [12]. This rhsTM binds to thrombin to form a thrombin-thrombomodulin complex that activates protein C, which in turn, plays an important role in regulating both coagulation and inflammation [13]. Although it was first approved as a therapeutic agent for disseminated intravascular coagulation (DIC) in Japan [14], previous reports demonstrated that it could also improve respiratory indexes and the mortality of acute respiratory distress syndrome (ARDS) with DIC [15, 16]. There are pathophysiological similarities between AE of IPF and ARDS, which are characterized by a pathological pattern of diffuse alveolar damage (DAD), and disordered coagulative state and excessive inflammation [4, 17]. Although research on the potential of rhsTM treatment for AE of IPF has reported some promising results, the effect of rhsTM treatment on AE of IPF remains unclear. Therefore, the aim of this systematic review was to summarize current accumulating evidence regarding benefits and harms of rhsTM treatment for AE of IPF.

## Methods

This study was reported following the Preferred Reporting Items for Systematic Reviews and Meta-Analyses (PRISMA) statement [18].

### Eligibility criteria

#### Subjects

Patients with AE of IPF were eligible for the review. Idiopathic NSIP was not included in this study because there has been no clear evidence that the prognosis of AE of idiopathic NSIP is the same as that of AE of IPF although it constitutes CFIP alongside of IPF [19]. IPF was diagnosed based on previously published joint statement of American Thoracic Society/European Respiratory Society/Japanese Respiratory Society/Latin American Thoracic Association (ATS/ERS/JRS/ALAT) or its recent update [10, 20]. Other types of idiopathic interstitial pneumonias (IIPs) such as cryptogenic organizing pneumonia (COP) and unclassifiable interstitial pneumonia were also excluded as they are known to demonstrate a different clinical course or might include undiagnosed IPs [1]. Similarly, secondary IPs such as CTD-IP and chronic hypersensitivity pneumonitis were also excluded due to different disease entities although AE of these diseases may pathophysiologically be similar to that of IPF [6, 7]. AE of IPF was diagnosed based on the previous report by Idiopathic Pulmonary Fibrosis Clinical Research Network [4] or the guideline published by the Japanese Respiratory Society [21]. Specifically, it was defined as worsening or development of dyspnoea manifested within 30 days, which is accompanied by newly emerging bilateral ground glass opacities and/or consolidation superimposed on underlying usual interstitial pneumonia (UIP) on high resolution computed tomography (HRCT) scan. Although heart failure or volume overload needed to be excluded for the diagnosis of the disease, other causes such as infection, pulmonary thromboembolism and aspiration were accepted as the triggering factors of this condition complying with the recent update of the diagnostic criteria for AE of IPF by the international working group [22]. AE at the first visit without any prior information of chronic IP was accepted if chronic interstitial changes of UIP were confirmed radiologically and/or pathologically and there were no apparent causes. Only the first episode of AE was considered for the review.

#### Intervention

The rhsTM (Asahi Kasei Pharma Corporation, Tokyo, Japan) was administered at a dose of 0.06 mg/kg/day or 380 U/kg/day for 6 days based on the approved dosage of the medicine [14, 23]. Conventional therapy included all of the other therapeutic options aside from rhsTM treatment such as corticosteroid, immunosuppressive drugs and anti-fibrotic agents. There were no restrictions regarding baseline therapy and the usage of other anticoagulants.

#### Outcome

The primary outcome of interest was all-cause mortality. The secondary outcomes of interest were disease-related mortality, oxygenation, discharge from a medical institution, health-related quality of life and adverse events such as bleeding and other coagulative abnormalities. The mortality was determined at 1 month, 3 months, 6 months and 1 year after the diagnosis of the disease. The 28-day or 30-day mortality and the 90-day mortality were designated as the 1-month mortality and the 3-month mortality, respectively.

#### Study type

Primary studies of any design except for a case report were eligible for the review if it compared a group receiving rhsTM treatment with a control group receiving conventional therapy without rhsTM treatment. Study designs were not limited to randomized controlled trials (RCTs) but observational studies were also included. There were no restrictions as to the number of included subjects and follow-up periods. Editorials, letters, review articles and conference proceedings were not considered. No language restrictions were implemented.

### Search strategy

Literature was searched through the following electronic databases, i.e., Medline (Ovid), EMBASE (Ovid), Science Citation Index Expanded (Web of Science), Cochrane Central Register of Controlled Trials (Cochrane Library), ClinicalTrials.gov, Google Scholar and ICHUSHI (the largest bibliographic database for medicine in Japan by Japan Medical Abstracts Society). Search was conducted on the 14th of August, 2019 and covered from 2002 as the current classification for IIPs was published in that year [24]. A string of search terms was constructed referring to previous reviews of the same topic [25, 26], which included ‘interstitial lung disease’, ‘acute exacerbation’ and ‘thrombomodulin’ (Additional file 1).

### Study selection and data extraction

Two reviewers (HK and OMP) independently screened retrieved records by titles and abstracts as well as full-texts if needed to select eligible reports. The same reviewers extracted relevant data similarly referring to a previous review of a similar subject [27]. These data included the first author’s name and publication year, study location, number of subjects and their demographic features in each treatment group, prior therapy and conjunctive treatment for AE, outcomes, number of subjects who developed the outcome, summary statistics and adverse events related to rhsTM treatment. Any disagreement in the selection of articles and data extraction was resolved by discussion between the reviewers. Authors were contacted to request for additional information if there was missing data for relevant outcomes.

### Risk of bias assessment

Two reviewers (HK and OMP) independently assessed risk of bias of individual studies using a tool called risk of bias in non-randomised studies of interventions (ROBINS-I) [28]. This tool is composed of 8 domains that are closely related to the bias of non-randomised studies. Each domain was rated as low, moderate, serious or critical and an overall risk of bias was determined based on the assessment of each domain.

### Statistical analysis

For binary outcomes the effect of rhsTM treatment was estimated as a risk ratio (RR), which was calculated based on the absolute number of the outcomes in each treatment group while continuous outcomes were summarized by a mean difference (MD). The results were statistically combined if there were three or more studies available that reported the same outcome. Meta-analysis was conducted by Mantel-Haenszel method using a fixed effect model on statistical software, Review Manager (RevMan) Version 5.3 (Copenhagen: The Nordic Cochrane Centre, The Cochrane Collaboration, 2014). Only univariate results were combined and multivariate results were reported qualitatively because variables in a multivariate model were to be diverse between studies. All results were reported with the 95% confidence interval (CI) and deemed statistically significant with *p*-value < 0.05. Heterogeneity between studies was estimated using the Q statistic and I^2^. Statistical significance of the test for heterogeneity was confirmed with *p*-value < 0.1 due to the low power of the test. I^2^ was interpreted as not important (< 30%), moderate (≥30 < 50%), substantial (≥50 < 70%) and considerable (≥70%) based on the Cochrane Handbook for Systematic Reviews of Interventions [29]. Subgroup analysis and sensitivity analysis were to be conducted focusing on study location (Asia/non-Asia) and studies with a similar risk of bias, respectively. Small study bias such as publication bias was to be examined graphically by a funnel plot and statistically by the Egger’s test if ten or more studies were available for meta-analysis [30].

## Results

### Selection of eligible studies

**Fig. 1:**
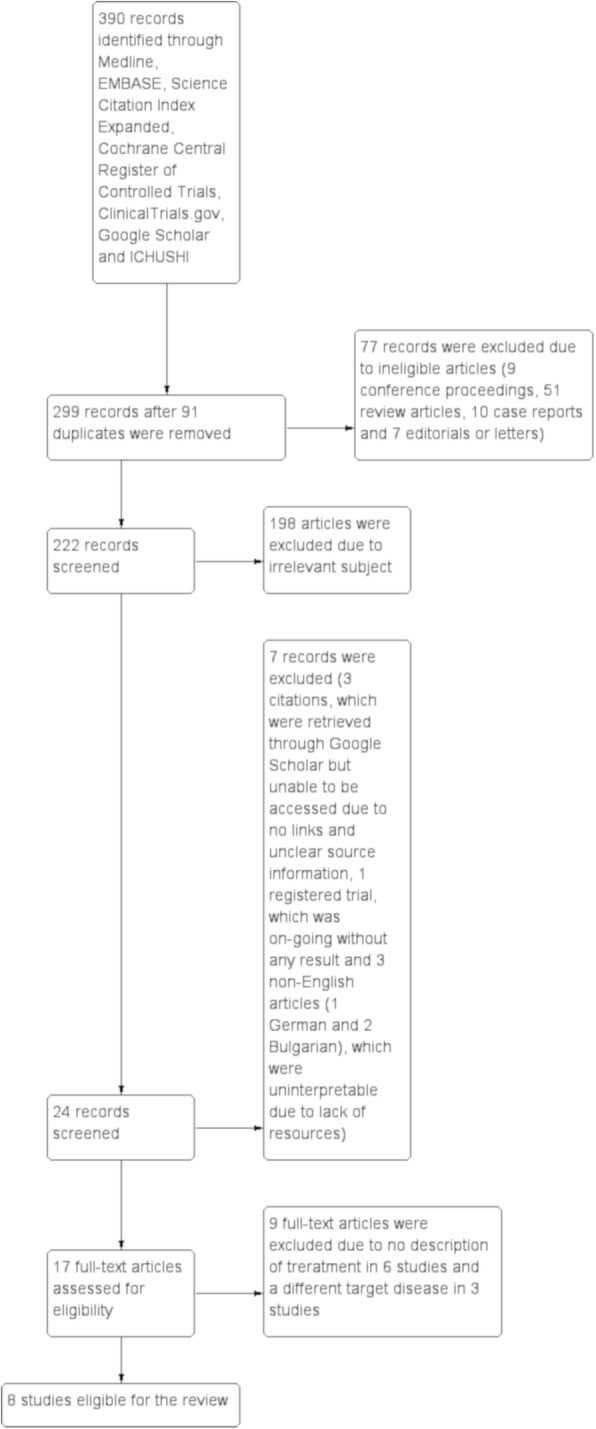
Study flow diagram. Out of a total of 390 records identified through a search of seven electronic databases, 299 records after removing 91 duplicates were screened by titles and abstract, which identified 77 ineligible records (9 conference proceedings, 51 review articles, 10 case reports and 7 editorials or letters) and 198 irrelevant reports. Out of the remaining 24 records, 7 records were excluded, which were composed of 3 records that were retrieved through Google Scholar but unable to be identified due to unavailable links and unclear source information, 1 registered trial that was on-going without any result available and 3 non-English articles (1 German and 2 Bulgarian) that were unable to be interpreted due to lack of resources. The remaining 17 articles were retrieved as full-texts and 9 of them were excluded due to no description of a specific treatment in 6 articles and a different target disease in 3 articles. Finally, eight reports/studies were deemed eligible for the review

Out of a total of 390 records identified through a search of seven electronic databases, eight reports/studies were first deemed eligible after excluding 91 duplicates, 77 ineligible records, 198 irrelevant articles and 16 other reports (Fig. 1). Two out of eight studies (Isshiki 2015 [31], Sakamoto 2018 [32]) were conducted by the same research group. Because the latter study was an extension from the former study, only the result of the latter report was considered for further analysis. One study (Arai 2019 [33]), which was composed of a total of 100 subjects including both IPF and non-IPF, was excluded from further analysis because non-IPF group did not undergo pathological diagnosis although all cases in the group were non-IPF IIPs according to the authors (personal communication). Additionally, two studies (Abe 2015 [34], Abe 2016 [35]) were also not considered for further analysis because the former study included idiopathic NSIP in conjunction with IPF and the latter study included radiologically possible UIP, which was not confirmed by surgical lung biopsy. As a result, the remaining four studies were focused for the review.

### Characteristics of included studies

All of the included studies were conducted by Japanese research groups. One study was a prospective trial (Hayakawa 2016 [36]) whereas the other studies were retrospective observations. All studies compared a group with rhsTM treatment with a historical control without rhsTM treatment (Sakamoto 2018 [32], Hayakawa 2016 [36], Tsushima 2014 [37], Kataoka 2015 [38]) (Table 1). Out of a total of 169 subjects included in the review, 95 subjects were allocated to rhsTM treatment while 74 subjects received conventional therapy without rhsTM treatment. The mean or median age of the treatment group ranged from 73.2 to 76.2 years while that of the control group was between 69.7 and 75 years. Over 80% of subjects were men in both groups. Baseline therapy before AE was reported in three studies (Sakamoto 2018 [32], Hayakawa 2016 [36], Kataoka 2015 [38]). In the rhsTM treatment group an anti-fibrotic agent (pirfenidone) was prescribed to 16 patients, corticosteroid to 14 patients and immunosuppressive drugs to 6 patients while in the control group 9, 13 and 3 patients received these medicines, respectively. The rhsTM was administered at a dose of 0.06 mg/kg/day for 6 days in three studies (Sakamoto 2018 [32], Tsushima 2014 [37], Kataoka 2015 [38]) while a dose of 380 U/kg/day was administered for 7 days in the remaining study (Hayakawa 2016 [36]). In one study (Kataoka 2015 [38]) the rhsTM treatment group was followed by low molecular weight heparin (LMWH) treatment for the median duration of 8 days while the control group received only LMWH treatment for the median of 10 days. In another study (Sakamoto2018 [32]) only six patients in the control group received LMWH treatment with a dose of 75 IU/kg/day for 14 days (no comparative data available between rhsTM treatment and LMWT). Other conjunctive therapeutic agents administered for AE included steroid pulse therapy for all patients and immunosuppressive agents for a total of 112 patients (Table 1). There was no significant difference in distributions of potential prognostic factors for AE of IPF between rhsTM treatment group and control group (Table 2).

**Table 1:** Characteristics of individual studies^a^

Study	Location	Design (comparison)	Number (n)	Age (years)	Gender (male) (n (%))	Pre-treatment (n)	Method of administering rhsTM	Other treatment for AE (n)	Anticoagulant for control group (n)
Sakamoto 2018 [32]	Japan	Retrospective (historical control)	45 vs. 35	Median (range) 75 (56–86) vs. 75 (59–82)	40 (88.9) vs. 28 (80.0)	Pir 9 vs. 7 Cs 11 vs. 9 IS 6 vs. 3	0.06 mg/kg/d for 6 days	mPSL all IS (CsA) 66	LMWH (75 IU /kg/d for 14 days) (6)
Hayakawa 2016 [36]	Japan	Prospective (historical control)	10 vs. 13	Mean (SD) 73.2 (9.5) vs. 69.7 (8.5)	8 (80.0) vs. 11 (84.6)	Pir 2 vs. 0	380 U/kg/d for 7 days	mPSL all IS 0 vs. 6	–
Tsushima 2014 [37]	Japan	Retrospective (historical control)	20 vs. 6	Mean (SE) 76.2 (0.4) vs. 73.7 (1.2)	14 (70.0) vs. 6 (100.0)	–	0.06 mg/kg/d for 6 days	mPSL all	–
Kataoka 2015 [38]	Japan	Retrospective (historical control)	20 vs. 20	Median (IQR) 73.5 (69.0–78.0) vs. 71.0 (64.8–78.0)	17 (85.0) vs. 19 (95.0)	Pir 5 vs. 2 Cs 3 vs. 4	0.06 mg/kg/d for median 6 days (range 3–6)	mPSL all IS (CsA) all	LMWH (75 IU/kg/d for median 10 days (range 7–22)) (20)

a, All comparisons are corresponding to rhsTM group vs. control group

*AE* acute exacerbation; *Cs* corticosteroid; *CsA* cyclosporine; *IS* immunosuppressive agents; *IQR* interquartile range; *LMWH* low molecular weight heparin; *mPSL* methylprednisolone pulse therapy; *pir* pirfenidone; *rhsTM* recombinant human soluble thrombomodulin; *SD* standard deviation; *SE* standard error;

**Table 2:** A comparison of potential prognostic factors for acute exacerbation of idiopathic pulmonary fibrosis between recombinant human soluble thrombomodulin (rhsTM) treatment group and control group^a^

Potential confounding factors/study	Sakamoto 2018 [32]	Hayakawa 2016 [36]	Tsushima 2014 [37]	Kataoka 2015 [38]
Pulmonary function at baseline				
%FVC	70.5 (35–114) vs 74.6 (30.5–112.8) (median (range))	68.1 ± 24.1 vs 58.6 ± 16.7	–	62.3 (51.4–70.1) vs 78.2 (53.9–84.9) (median (IQR))
%DLCO	49.2 (23.8–110.2) vs 55.4 (26.3–75.6) (median (range))	–	–	45.8 (31.0–50.9) vs 36.5 (29.4–51.8) (median (IQR))
Laboratory data at onset of AE				
WBC	10,500 (4700–24,000) vs 10,200 (3300–16,900) (/mm^3^) (median (range))	–	14,000 ± 462 vs 15,820 ± 291 (/uL) (mean ± SE)	10,350 (8550–12,775) vs 10,350 (7175–12,225) (/mm^3^) (median (IQR))
LDH	353 (232–593) vs 351 (193–1005) (IU/L) (median (range))	378 ± 118 vs 444 ± 173 (IU/L)	782 ± 41 vs 477 ± 13 (IU/L) (mean ± SE)	300 (273–353) vs 313 (277–387) (IU/L) (median (IQR))
KL-6	1299 (286–10,469) vs 1038 (483–4660) (U/mL) (median (range))	1512 ± 583 vs 2060 ± 1520 (U/mL)	1812 ± 111 vs 1956 ± 218 (U/mL) (mean ± SE)	1278 (966–1869) vs 1255 (945–1496) (U/mL) (median (IQR))
SP-D	288 (177–1070) vs 317 (155–1410) (ng/mL) (median (range))	482 ± 527 vs 676 ± 711 (ng/mL)	–	202 (167–381) vs 203 (143–260) (pg/mL) (median (IQR))
P/F ratio	256 (61–380) vs 260 (74–418) (median (range))	168 ± 56 vs 183 ± 47	107 ± 3.9 vs 121 ± 5.9 (mmHg) (mean ± SE)	224 (199–247) vs 238 (195–266) (mmHg) (median (IQR))
CRP	6.0 (0.6–20.4) vs 6.9 (0.1–32.5) (mg/dL) (median (range))	11.5 ± 8.3 vs 11.0 ± 11.1 (mg/dL)	13.1 ± 0.7 vs 11.8 ± 0.5 (mg/mL) (mean ± SE)	4.7 (3.4–9.4) vs 6.8 (2.2–10.5) (mg/dL) (median (IQR))

a, All comparisons are corresponding to rhsTM treatment group vs. control group. The values are expressed as mean ± standard deviation unless otherwise specified. There was no statistically significant difference in distributions of any potential prognostic factors between the two comparative groups

*AE* acute exacerbation; *CRP* C-reactive protein; *IQR* interquartile range; *KL-6* Krebs von den Lungen-6; *LDH* lactate dehydrogenase; *%DLCO* percentage of predicted diffusing capacity of the lung for carbon monoxide; *%FVC* percentage of predicted forced vital capacity; *P/F* partial pressure of arterial oxygen to fraction of inspired oxygen; *rhsTM* recombinant human soluble thrombomodulin; *SE* standard error; *SP-D* surfactant protein-D; *WBC* white blood cell;

### Risk of bias in individual studies

An overall risk of bias was deemed low in one study (Kataoka 2015 [38]) whereas that of the other three studies was rated as serious (Sakamoto 2018 [32], Hayakawa 2016 [36], Tsushima 2014 [37]). This different finding was largely due to the bias assessment of confounding domain, which was sufficiently addressed using a propensity score in the former study. However, it was inadequately explained or not considered at all in the other three studies (Table 3).

**Table 3:** Risk of bias in individual studies

study	confounding	selection of participants	classification of intervention	deviations from intended interventions	missing data	measurement of outcomes	selection of the reported result	overall risk of bias
Sakamoto2018 [32]	**Serious**	Low	Low	Low	Low	Low	Low	**Serious**
Hayakawa 2016 [36]	**Serious**	Low	Low	Low	Low	Moderate	Moderate	**Serious**
Tsushima 2014 [37]	**Serious**	Moderate	Low	Low	Low	Low	Low	**Serious**
Kataoka 2015 [38]	Low	Low	Low	Low	Low	Low	Low	Low

Text in bold indicates serious risk of bias

### Effect of recombinant human soluble thrombomodulin (rhsTM) treatment

#### Univariate analysis

All four studies reported all-cause mortality using univariate analysis, which was described as 28-day mortality in two studies, 3-month mortality in three studies and 1-year mortality in one study (Table 4).

**Table 4:** Summary of the effect of recombinant human soluble thrombomodulin (rhsTM) treatment^a^

Study	Oxygenation (improvement of PaO2/FiO2 ratio (mmHg))	All-cause mortality						Adverse effects (n)
		Count of death (rhsTM) (n (%))	Count of death (control) (n (%))	Risk ratio (95% CI)	Multivariate (95% CI)	time at measurement	Adjusted factors	
Sakamoto 2018 [33]^b^	–	15 (33.4)	22 (62.9)	**0.53 (0.33–0.86)**	**OR 0.25 (0.09–0.68)**	3 months	No description	Haemoptysis and haematuria (1)
Hayakawa 2016 [37]^b^	**MD 21.0 (95% CI: 12.9–29.1)**	3 (30) 4 (40) 6 (60)	6 (46) 11 (85) 12 (92)	0.65 (0.21–1.98) 0.47 (0.21–1.05) 0.65 (0.38–1.10)	- -	28 days 3 months 1 year	–	Not side effects
Tsushima 2014 [37]	–	7 (35.0)	5 (83.0)	**0.42 (0.21–0.84)**	–	28 days	–	–
Kataoka 2015 [38]	–	6 (30.0)	13 (65.0)	**0.46 (0.22–0.97)**	**OR 0.21 (0.05–0.91)**	3 months	propensity score^c^	Haemosputum (1) DVT (1)

a, All comparisons are corresponding to rhsTM treatment group vs. control group and significant results are indicated in boldface; b, An absolute number of the outcome was calculated from the percentage data available; c, A propensity score was calculated adjusting for age, sex, PaO2/FiO2 ratio, respiratory rate, APACHE II score, WBC, CRP, LDH, KL-6, SP-D and D-dimer;

*APACHE II score* acute physiology and chronic health evaluation II score; *CI* confidence interval; *CRP* C-reactive protein; *DVT* deep venous thrombosis; *HR* hazard ratio; *KL-6* Krebs von den Lungen-6; *LDH* lactate dehydrogenase; *MD* mean difference; *OR* odds ratio; *PaO2/FiO2* partial pressure of arterial oxygen to fraction of inspired oxygen; *rhsTM* recombinant human soluble thrombomodulin; *SP-D* surfactant protein-D; *WBC* white blood cell;

The 1-month all-cause mortality was significantly better in rhsTM treatment group than control group with an RR of 0.42 (95% CI: 0.21–0.84) in one study (Tsushima 2014 [37]) whereas there was no statistically significant difference with an RR of 0.65 (95%CI: 0.21–1.98) in the other study (Hayakawa 2016 [36]). The mortality was 35.0 and 30% in rhsTM treatment group whereas it was 83.0 and 46% in control group in these two studies, respectively (Table 4).

**Fig. 2:**
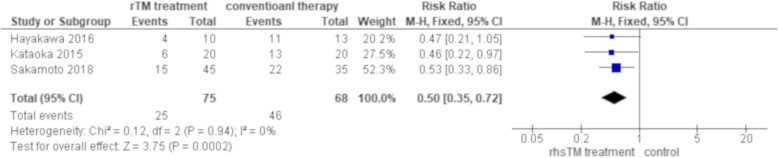
3-month all-cause mortality. A total of three studies reported 3-month all-cause mortality using univariate analysis, which included 143 subjects. A total of 25 out of 75 patients (33.3%) in rhsTM treatment group died within 3 months while 46 out of 68 patients (67.6%) in control group died within the same period of time. A pooled analysis of three studies demonstrated that 3-month all-cause mortality was significantly better in rhsTM treatment group than control group with an RR of 0.50 (95% CI: 0.35–0.72) with no heterogeneity (chi^2^ = 0.12, df = 2, *p* = 0.94, I^2^ = 0)

The 3-month all-cause mortality was significantly better in rhsTM treatment group than control group in two studies (Sakamoto 2018 [32], Kataoka 2015 [38]) whereas there was no statistically significant difference in the other study (Hayakawa 2016 [36]). The mortality ranged from 30.0 to 40% in rhsTM treatment group whereas it was between 62.9 and 85% in control group (Table 4). A pooled analysis of three studies yielded a statistically significant result with an RR of 0.50 (95% CI: 0.35–0.72) with no heterogeneity (chi^2^ = 0.12, df = 2, *p* = 0.94, I^2^ = 0) (Fig. 2).

There was no statistically significant difference of 1-year mortality between rhsTM treatment group and control group in one study (Hayakawa 2016 [36]). A total of 6 out of 10 patients (60%) in rhsTM treatment group died within 1 year whereas 92% of 13 patients died in control group, which yielded an RR of 0.65 (95% CI: 0.38–1.10) (Table 4).

Although subgroup analysis and sensitivity analysis could not be performed due to a small number of included studies, one study with low risk of bias demonstrated an improvement of 3-month all-cause mortality with an RR of 0.46 (95% CI: 0.22–0.97) (Kataoka 2015 [38]), which was similar to the results of the other two studies with serious risk of bias that demonstrated RRs of 0.53 (95% CI: 0.33–0.86) and 0.47 (95% CI: 0.21–1.05) (Sakamoto 2018 [32], Hayakawa 2016 [36]), respectively (Table 4).

Another outcome of interest reported was a change in the respiratory index in one study (Hayakawa 2016 [36]). There was a statistically significant difference of an improvement in the partial pressure of arterial oxygen to fraction of inspired oxygen (PaO2/FiO2) ratio on day 8 after commencing the rhsTM treatment with an MD of 21.0 (95% CI: 12.9–29.1) (Table 4).

Small study bias such as publication bias could not be evaluated due to a small number of included studies.

#### Multivariate analysis

Two studies reported all-cause mortality using multivariate analysis. A propensity score was employed to adjust for confounding factors in one study (Kataoka 2015 [38]) whereas there was no explanation of adjusted factors in another study (Sakamoto 2018 [32]). The rhsTM treatment significantly improved 3-month all-cause mortality with odds ratios (ORs) of 0.21 (95% CI: 0.05–0.91) and 0.25 (95% CI: 0.09–0.68) in these two studies (Kataoka 2015 [38], Sakamoto 2018 [32]), respectively (Table 4).

#### Adverse events

Adverse events related to rhsTM treatment were described in three studies including one study, in which no side effects were detected (Hayakawa 2016 [36]). There were one case each of haemosputum and deep venous thrombosis in one study (Kataoka 2015 [38]), both of which were improved after discontinuation or change of the treatment. Another study (Sakamoto 2018 [32]) reported a patient with both haemoptysis and haematuria, which resolved spontaneously. No severe bleeding or coagulopathy were reported (Table 4).

## Discussion

This systematic review and meta-analysis demonstrated beneficial effects of rhsTM treatment on 3-month all-cause mortality of AE of IPF based on combined results of univariate analysis although its effect on 1-month mortality was inconsistent between studies. This finding was confirmed by the results of multivariate analysis in all studies that reported it. Furthermore, oxygenation was significantly improved after rhsTM treatment although it was reported in only one study. Finally, rhsTM treatment was considered safe and tolerable as only a few cases of minor bleeding and one thrombotic event were developed.

AE of IPF is characterised pathologically by damage to alveolar epithelial cells and surrounding capillary vasculature, i.e., DAD superimposed on chronic fibrotic interstitial changes [39]. DAD is not unique to AE of IPF but recognized as a common pathological finding manifested in severe respiratory illnesses resulting from intrinsic or extrinsic excessive insult to the lung such as ARDS [40]. Although the exact pathogenesis of AE of IPF remains unclear, it is reported to involve massive inflammatory reactions as well as disordered coagulation and fibrinolysis that lead to the characteristic hyaline membrane formation in DAD [39]. Thrombomodulin is a transmembrane glycoprotein present on the endothelial cell surface of the body and plays a pivotal role in regulating coagulation cascade [41]. It binds to thrombin to form thrombin-thrombomodulin complex in the blood vessel and enhances the activation of protein C, which in turn, suppresses the activated coagulation factor V and VIII with the aid of protein S and reduces further thrombin formation to control coagulative state [41]. As activated protein C possesses anti-inflammtory, cytoprotective and anti-apoptotic properties through the modulation of the transcription of pro-inflammatory mediators [42], thrombomodulin also indirectly involves host defence mechanism through this pathway [43]. Furthermore, thrombomodulin expresses anti-inflammation irrespective of the protein C pathway. It directly binds to high motility group box 1 (HMGB1) secreted by inflammatory cells and Lewis Y antigen in lipopolysaccharide on gram-negative bacteria [44], by which it prevents these molecules from interacting with their own receptors and suppresses the signal transduction to the down-stream pro-inflammatory pathway [45]. As an elevation of serum HMGB1 was noted in patients with acute lung injury [46] and a higher level of the molecule is reported to predict shorter survival of patients with AE of IPF [47], thrombomodulin, which plays a role in controlling the effect of HMGB1, may be essential in the pathogenesis of the disease.

Previous studies demonstrated that thrombomodulin is excessively secreted into bloodstream in severe illnesses such as sepsis and ARDS [48, 49]. Thrombomodulin was also reported to be elevated in bronchoalveolar lavage fluid (BALF) and shown to correlate with the prognosis of AE of IP [50]. This soluble thrombomodulin molecule detached from the endothelial cell surface may be unduly generated by proteolytic cleavage of the protein, which is mediated by a wide range of proteases augmented under these critical circumstances [48]. The soluble form of thrombomodulin may lack its full-brown property due to the breakdown of the molecule at various dimensional sites although some previous studies reported that it might remain functionally active [51]. However, at least the soluble thrombomodulin may insufficiently activate protein C due to little engagement of endothelial protein C receptor that is noted to boost the reaction on the endothelial cell surface [43]. Furthermore, it is reported that the transcription of thrombomodulin is suppressed and the expression of the molecule on the endothelium is reduced in severe clinical conditions such as ARDS and AE of IPF [48, 52]. As a result, accelerated inflammatory reactions in conjunction with unprotected thrombotic state would continue to injure respiratory system as well as other organs in these critical conditions.

The rhsTM was constructed through a cloning technique, which consists of extracellular domains of native thrombomodulin [12]. Since it carries anti-inflammatory and anti-coagulative property, it is expected to ameliorate both disintegrated inflammation and coagulation manifested in systemic inflammatory disorders and improve the prognosis of the disease [13]. The rhsTM treatment was first approved for clinical use in Japan based on the result of a small trial that depicted beneficial effects of rhsTM treatment on DIC triggered by hematologic malignancy and sepsis [14]. However, a similar efficacy of rhsTM treatment for sepsis-associated coagulopathy was not confirmed in a recent RCT that was conducted by international multi-institutions [53]. In addition, the latest systematic review and meta-analysis did not reveal the advantage of rhsTM treatment for sepsis-induced DIC [54]. Conversely, only a small number of studies reported the effect of rhsTM treatment on DIC complicated with ARDS. One study reported an improvement of lung injury score with rhsTM treatment [15] and another study demonstrated improved in-hospital mortality [16]. However, all of these studies were of retrospective design and no RCTs have been conducted to elucidate the efficacy of rhsTM treatment for ARDS. Although this current review demonstrated that rhsTM treatment could improve all-cause mortality of AE of IPF, it was based on a small number of low-quality studies. In short, a high-quality study reported non-significant results of rhsTM treatment for sepsis-associated coagulopathy [53] whereas a small number of low-quality studies demonstrated its possibly anecdotal beneficial effects on ARDS and AE of IPF [15, 16]. Therefore, clinicians should refrain from implementing rhsTM treatment in their daily practice. Furthermore, the recombinant human activated protein C (rhAPC), which formerly emerged as a promising medicine for sepsis or acute lung injury, was eventually removed from the market due to lack of beneficial effects confirmed by a large-scale RCT [55]. Biologically the mechanism of action of rhAPC constitutes the properties of rhsTM treatment [43]. Therefore, it may be too soon to be optimistic that rhsTM treatment is beneficial for AE of IPF although it seems to be a reasonable therapeutic option, in particular, under the current suffocating circumstances where there has been no established effective treatment for AE of IPF [10].

There are some of limitations that need to be kept in mind to appropriately interpret the findings of this review. Firstly, this review was composed of only four studies and most of these studies included a small sample with less than 50 subjects. Two studies (Arai 2019 [33], Abe 2016 [35]) were excluded from detailed analysis because both of these studies included IIPs with possible UIP pattern on HRCT without surgical lung biopsy, which possibly might have led to the inclusion of non-IPF cases. Therefore, the reports may be anecdotal and small study bias is likely to exist although it could not be evaluated due to a small number of included studies. However, prognosis of AE of non-IPF IIPs and AE of other  fibrotic interstitial lung diseases is similarly poor to that of AE of IPF [19, 33]. The rhsTM treatment might be effective for AE of non-IPF IIP cases as was suggested in the report of Arai et al. [33] although additional trials are needed for a definitive conclusion. Secondly, only one study in this review was a prospective trial whereas the other studies were retrospective observations. In addition, the use of historical control employed in all studies is a major concern. Although a propensity score was introduced in one study to adjust for baseline differences, there remains a certain factor that may impact the prognosis of the disease but cannot be statistically handled, e.g., a change of care for patients with AE of IPF over time. In fact, the mortality of a control group in individual studies of this review was substantially high with 3-month mortality ranging from 62.9 to 85% in comparison to 40% in a recent report [9]. Therefore, a study design with a historical control may have distorted the true effect of rhsTM treatment. Thirdly, only two studies conducted multivariate analysis. As a result, most of the studies constituting this review were rated as serious in risk of bias assessment. Lastly, all studies in this review were conducted in Japan. As a result, the generalizability of the findings is limited. In particular, as Japanese patients are reported to be more susceptible to drug-induced IP [56] and other types of rapidly progressive IP [57], the effect of rhsTM treatment may vary depending on the ethnic background. Therefore, a large-scale international RCT is necessary to address these limitations and confirm the efficacy of rhsTM treatment for AE of IPF. To this end, one randomized placebo-controlled trial was undertaken in Japan and recently completed (NCT02739165) [58]. The result of this trial is expected to provide a conclusive finding. However, the true effect of rhsTM treatment may not be clarified due to its small sample size, i.e., only 74 subjects in total. In a previous trial of rhAPC for ARDS that enrolled around 70 subjects, the authors mentioned that underpowering could not be excluded to explain their negative result [59] and a larger trial with around 1600 subjects was required to obtain a definitive conclusion [55]. Therefore, whatever the result will be in this recent RCT, a larger-scale international trial will be required to confirm the finding.

## Conclusion

In this systematic review and meta-analysis, the rhsTM treatment was demonstrated to improve 3-month all-cause mortality of AE of IPF. It was considered safe and tolerable with only minor adverse effects. However, these findings need to be interpreted with caution due to a small number of studies, serious risk of bias and limited generalizability.

## Supplementary information

**Additional file 1.** Search terms for each electronic database.

## Data Availability

The dataset used and/or analyzed during the current study will be available from the corresponding author on a reasonable request after the final result is published in a journal.

## Abbreviations

AE: Acute exacerbation
ARDS: Acute respiratory distress syndrome
BALF: Bronchoalveolar lavage fluid
CFIP: Chronic fibrosing interstitial pneumonia
CI: Confidence interval
COP: Cryptogenic organizing pneumonia
CTD: Connective tissue disease
DAD: Diffuse alveolar damage
DIC: Disseminated intravascular coagulation
HMGB1: High motility group box 1
HR: Hazard ratio
HRCT: High resolution computed tomography
IIP: Idiopathic interstitial pneumonia
IP: Interstitial pneumonia
IPF: Idiopathic pulmonary fibrosis
LMWH: Low molecular weight heparin
MD: Mean difference
NSIP: Non-specific interstitial pneumonia
OR: Odds ratio
PaO2/FiO2: Partial pressure of arterial oxygen to fraction of inspired oxygen
PRISMA: Preferred reporting items for systematic review and meta-analyses
RCT: Randomized controlled trial
rhAPC: Recombinant human activated protein C
rhsTM: Recombinant human soluble thrombomodulin
ROBINS-I: Risk of bias in non-randomised studies of interventions
RR: Risk ratio
UIP: Usual interstitial pneumonia
