# Supplementary material for: The efficacy of recombinant human soluble thrombomodulin (rhsTM) treatment for acute exacerbation of idiopathic pulmonary fibrosis: a systematic review and meta-analysis
Source: BMC Pulm Med. 2020 Mar 2;20:57. doi: 10.1186/s12890-020-1092-3 (PMC7053075; doi:10.1186/s12890-020-1092-3)
Supplement: Supplementary file 1 — Additional file 1. Search terms for each electronic database. [file 12890_2020_1092_MOESM1_ESM.docx]

Search terms for each electronic database

Medline (Ovid)

1 exp Lung Diseases, Interstitial/

2 exp Pulmonary Fibrosis/

3 (interstitial adj3 lung adj3 disease$).mp.

4 (interstitial adj3 pneumoni$).mp.

5 alveolitis.mp.

6 (pulmonary adj3 fibros$).mp.

7 exp Disease Progression /

8 (acute adj3 exacerbation?).mp.

9 (disease adj3 progression?).mp.

10 (disease adj3 exacerbation?).mp.

11 (deterioration?).mp.

12 exp Thrombomodulin/

13 thrombomodulin.mp.

14 ART-123.mp.

15 recomodulin.mp.

16 1 or 2 or 3 or 4 or 5 or 6

17 7 or 8 or 9 or 10 or 11

18 12 or 13 or 14 or 15

19 16 and 17 and 18

20 limit 19 to yr="2002 -Current"

EMBASE (Ovid)

1 exp interstitial lung disease/

2 exp lung fibrosis/

3 (interstitial adj3 lung adj3 disease$).mp.

4 (interstitial adj3 pneumoni$).mp.

5 alveolitis.mp.

6 (pulmonary adj3 fibros$).mp.

7 exp disease exacerbation /

8 exp deterioration /

9 (acute adj3 exacerbation?).mp.

10 (disease adj3 progression?).mp.

11 (disease adj3 exacerbation?).mp.

12 exp thrombomodulin/ or exp recombinant thrombomodulin/

13 thrombomodulin.mp.

14 ART-123.mp.

15 recomodulin.mp.

16 1 or 2 or 3 or 4 or 5 or 6

17 7 or 8 or 9 or 10 or 11

18 12 or 13 or 14 or 15

19 16 and 17 and 18

20 limit 22 to yr="2002 -Current"

Cochrane Central Registry of Controlled Trials (CENTRAL)

#1 MeSH descriptor: [Lung Diseases, Interstitial] explode all trees

#2 MeSH descriptor: [Pulmonary Fibrosis] explode all trees

#3 interstitial near/3 lung near/3 disease*:ti,ab,kw

#4 interstitial near/3 pneumoni*:ti,ab,kw

#5 alveolitis:ti,ab,kw

#6 pulmonary near/3 fibros*:ti,ab,kw

#7 MeSH descriptor: [Disease Progression] explode all trees

#8 MeSH descriptor: [Clinical Deterioration] explode all trees

#9 acute near/3 exacerbation?:ti,ab,kw

#10 disease near/3 progression?:ti,ab,kw

#11 disease near/3 exacerbation?:ti,ab,kw

#12 deterioration?:ti,ab,kw

#13 MeSH descriptor: [Thrombomodulin] explode all trees

#14 thrombomodulin:ti,ab,kw

#15 ART-123:ti,ab,kw

#16 recomodulin:ti,ab,kw

#17 #1 or #2 or #3 or #4 or #5 or #6

#18 #7 or #8 or #9 or #10 or #11 or #12

#19 #13 or #14 or #15 or #16

#20 #17 and #18 and #19

#21 #20 with Publication Year from 2002 to 2019

Science Citation Index Expanded (Web of Science)

#1 TS=("interstitial NEAR/3 disease$") OR TS=("interstitial NEAR/3 pneumoni*") OR TS=(alveolitis) OR TS=("pulmonary NEAR/3 fibros*")

#2 TS=(acute NEAR/3 exacerbation$) OR TS=(disease NEAR/3 progression$) OR TS=(disease NEAR/3 exacerbation$) OR TS=(deterioration$)

#3 TS=(thrombomodulin) OR TS=(ART-123) OR TS=(recomodulin)

#4 #1 AND #2 AND #3

#5 #4 AND (2002-2019)

Google Scholar

(“interstitial lung disease” OR “usual interstitial pneumonia” OR “idiopathic pulmonary fibrosis”) (“acute exacerbation” OR "disease progression" OR "disease exacerbation") (thrombomodulin OR ART-123 OR recomodulin)

ClinicalTrials.gov

# ART-123

ICHUSHI (Japan Medical Abstracts Society)

#1肺疾患-間質性/TH or 間質性肺疾患/AL

#2肺炎-間質性/TH or 間質性肺炎/AL

#3肺線維症/TH or 肺線維症/AL

#4肺炎-特発性間質性/TH or 特発性間質性肺炎/AL

#5肺線維症-特発性/TH or 特発性肺線維症/AL

#6 臨床的増悪/TH or 急性増悪/AL

#7 Thrombomodulin/TH or トロンボモジュリン/AL

#8 “Thrombomodulin Alfa”/TH or ART-123/AL

#9 “Thrombomodulin Alfa”/TH or リコモジュリン/AL

#10 #1 or #2 or #3 or #4 or #5

#11 #7 or #8 or #9

#12 #6 and #10 and #11

#13 #12 and (PT=会議録除く)

#14 #13 and (PT=症例報告・事例除く)

#15 #14 and (PT=原著論文)

#16 #15 and (DT=2002:2019)
